# The Osteoclast in Bone Metastasis: Player and Target

**DOI:** 10.3390/cancers10070218

**Published:** 2018-06-27

**Authors:** Antonio Maurizi, Nadia Rucci

**Affiliations:** Department of Biotechnological and Applied Clinical Sciences, University of L’Aquila, 67100 L’Aquila, Italy; antoniomaurizi@outlook.com

**Keywords:** breast cancer, bone metastases, osteoclasts, antiresorptive drugs, vicious cycle

## Abstract

Bone metastases are frequently the final fate of breast and prostate cancer patients. According to the definition of metastasis as an incurable disease, to date there are no effective treatments for tumor-associated bone metastases and this represents a real challenge for the researchers in the field. The bone is a heterogeneous environment that represents a fertile soil for tumor cells, supporting their growth. Among the different cell types present in the bone, in this review we will focus our attention on the osteoclasts, which are crucial players in the so called “vicious cycle”, a phenomenon triggered by tumor cells eventually leading to both tumor proliferation as well as bone deregulation, thus fueling the development of bone metastasis. The complex network, linking tumor cells to the bone by activating osteoclasts, represents a fruitful target for the treatment of bone metastases. In this review we will describe how tumor cells perturb the bone microenvironment by actively influencing osteoclast formation and activity. Moreover, we will describe the current antiresorptive drugs employed in the treatment of bone metastases as well as new, targeted therapies able to affect both cancer cells and osteoclasts.

## 1. Bone Metastases: Pathological Features

Metastasis is, so far, an incurable disease. The skeleton is one of the most frequent sites of metastases, behind the lungs and liver [1]. Prostate and breast cancers are responsible for the majority of bone metastases. Indeed, in prostate cancer the bone is often the unique site of metastasis, with a prevalence of up to 90%, while for breast cancer the prevalence is around 65%–75%. Notably, the median survival of breast cancer patients who develop bone metastases is twenty months, while it dramatically drops to six months in case of lung metastases and further to three months in patients experiencing liver metastases [2]. The median survival of prostate cancer patients with bone metastases is better, at around 53 months [3]. Therefore, in breast and prostate cancers, those patients with unique metastases in the bone represent as a distinct subset of metastatic patients with a better prognosis than those with liver or lung metastases. Moreover, in bone-only metastatic patients there is usually a multifocal dislocation of the lesions, which mainly affect the sternum, pelvis and lumbar spine [4]. Unfortunately, almost 50% of breast cancer patients with bone metastases are destined also to develop metastases in soft tissues.

Patients with bone metastases experience a very miserable quality of life, due to severe pain, spinal cord compression, fractures, bone marrow aplasia and hypercalcemia, the latter being the principal cause of death. In fact, hypercalcemia can induce gastrointestinal dysfunctions, as well as constipation, polyuria and fatigue. In the advanced stage, it is the leading cause of renal failure and cardiac arrhythmias [5]. All these symptoms are usually classified as skeletal-related events (SREs).

Despite some cases of bone metastases being asymptomatic and causing damage to bone structure without causing pain [6], the development of bone pain is usually highly indicative of bone metastases [7]. The pain associated with bone metastases could be of inflammatory or mechanical origin; inflammatory pain is due to the local release of cytokines and chemical mediators by the tumor cells, thus stimulating intraosseous nerves. Mechanical pain is caused by the pressure exerted by tumor mass within the bone, associated with loss of bone strength. Although they are not effective in increasing survival, current antiresorptive agents and radiotherapy reduce bone pain, thus significantly improving patient’s quality of life [8].

At diagnosis, bone metastases can be classified, according to their radiological appearance, as osteolytic, osteosclerotic or mixed, the latter when both features are present in the same region. Osteolytic lesions are preferentially caused by breast cancer cells and are characterized by the complete destruction of bone and its substitution with a tumor mass. Since the bone is a hard tissue, this destruction seems to be necessary for tumor cells to make space for their growth. The first theories explained bone destruction as the result of a physical compression caused by tumor cell growth, while other scientists hypothesized an acquired capacity of tumor cells to reabsorb the bone. Indeed, over the years a univocal *consensus* has been reached on the fact that the exacerbated bone resorption is the result of the ability of tumor cells to induce osteoclastogenesis, that is the formation of the cells devoted to resorbing bone [9].

Osteosclerotic (i.e., osteoblastic) metastases are instead characterized by apposition of new bone of poor quality that is secreted by the osteoblasts because of a conditioning by tumor cells. Moreover, osteosclerotic metastases are usually preceded, and likely triggered, by an exacerbated osteoclast activation. This also explains why the current antiresorptive therapies are effective also in this type of bone metastasis.

Whichever histopathological kind of bone metastases, all are determined by a deregulation of bone homeostasis caused by tumor cells.

## 2. Bone Physiology

As described by Stephen Paget more than 100 years ago with the “seed and soil” theory [10], tumor cells can only root themselves in those distant organs where they find the appropriate conditions to grow, thus explaining why breast cancer cells preferentially metastasize the bone. This tissue has a peculiarity that is the ability to continuously renew itself through a physiological process called bone remodeling. This is important to guarantee good quality of the bone, the repair of microfractures and the homeostasis of calcium [11].

### 2.1. Bone Remodeling: The Virtuous Cycle

Bone cells, that is osteocytes, osteoblasts and osteoclasts, actively participate in the cycle of bone remodeling, following space and time well-controlled steps, as described:

Activation phase. The starting point of bone remodeling is triggered by physiological and/or pathological stimuli. Osteocytes, the bone cells buried in the bone matrix, having mechano-sensorial functions, can promote the activation phase following the perception of changes in mechanical loading. Other inputs can come from micro-fractures or the release of cytokines, like insulin-like growth factor (IGF), tumor necrosis factor (TNF) α, parathyroid hormone (PTH) and interleukin (IL) 6, which in turn promote the detachment of the lining cells (i.e., quiescent osteoblasts) from the bone surface and the exposition of the latter for the subsequent step of erosion [12]. During this phase there is also attraction of osteoclast precursors from the blood and their fusion and differentiation into multinucleated osteoclasts.

Resorption phase. Polarized multinucleated osteoclasts firmly adhere to the bone surface by the so-called sealing zone and start to dissolve the bone. This phase is relatively fast compared to the others of the cycle of remodeling, lasting 2 weeks. It ends because of the activation of osteoclast apoptosis, thus ensuring that excess resorption does not occur.

Reverse phase. This phase is so named because of the involvement of reverse cells, whose role has not yet been completely clarified. These are macrophage-like cells with a likely function of removal of debris produced during bone degradation, thus preparing the bone for the anabolic action of osteoblasts. Moreover, these cells receive signals that allow the coupling of bone resorption bone formation activities [13,14].

Formation phase. As a consequence of bone resorption, there is the release of several factors usually stored in the bone matrix, including bone morphogenetic proteins (BMPs), fibroblast growth factors (FGFs), and transforming growth factor (TGF) β, which are chemoattractants for osteoblasts in the reabsorbed area. This is the longest phase of the remodeling, lasting 4 months, during which the osteoblasts produce new bone matrix, initially not calcified (osteoid) and then allow its mineralization, through the deposition of hydroxyapatite crystals amongst the collagen fibrils. This is a well-controlled process relying on a fine tune regulation of the local calcium and phosphate concentrations and the ratio of inorganic pyrophosphate to phosphate [15]. The complete formation and mineralization of the previously resorbed bone matrix leads the osteoblasts to the following possible fates: undergo programmed cell death, become a bone-lining cell or remain “buried alive” within the bone matrix thus becoming osteocytes.

Correct bone homeostasis strictly relies on a perfect equilibrium between osteoblast and osteoclast functions. Deregulation of osteoclast activity is a key event in the development of bone metastases, therefore in the next paragraph we will illustrate in more detail the physiology of this cell, and how it has become the best therapeutic target for the treatment of bone metastases.

### 2.2. Biology of the Osteoclast

**Figure 1:**
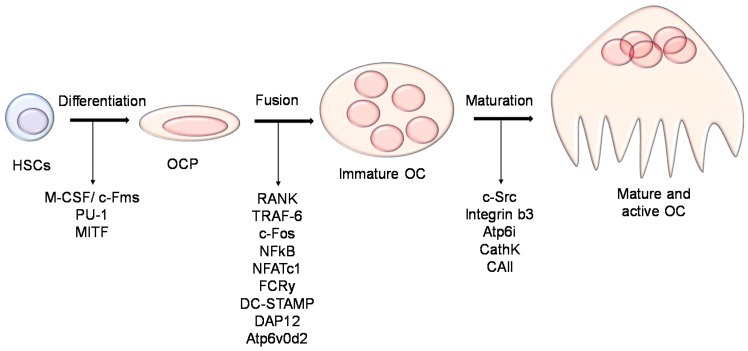
Osteoclast formation and differentiation. Cartoon illustrating the different steps and molecules involved in the osteoclast differentiation from the hematopoietic stem cells towards the mature osteoclast. HSCs: hematopoietic stem cells; OCP: osteoclast precursor; OC: osteoclast.

Osteoclasts are multinucleated cells that resorb the bone matrix keeping the skeleton healthy by ensuring bone turnover [16]. They arise from the monocyte-macrophage lineage under the stimulation of two pivotal cytokines: macrophage-colony stimulating factor (M-CSF) and receptor activator of nuclear factor kappa-β ligand (RANKL), both mainly produced by osteoblasts in the bone, thus actively participating in the regulation of osteoclast formation. Osteoclast differentiation transpires to be a complex process that can be divided in three major steps: (1) the commitment of the hematopoietic stem cells (HSCs) towards the macrophages lineage; (2) the acquisition of the positivity for the tartrate resistant acid phosphatase (TRAcP) enzyme and calcitonin receptor, thus giving rise to an osteoclast precursor and (3) the fusion of the osteoclast precursors eventually forming to mature polynucleated osteoclasts (Figure 1).

**Figure 2:**
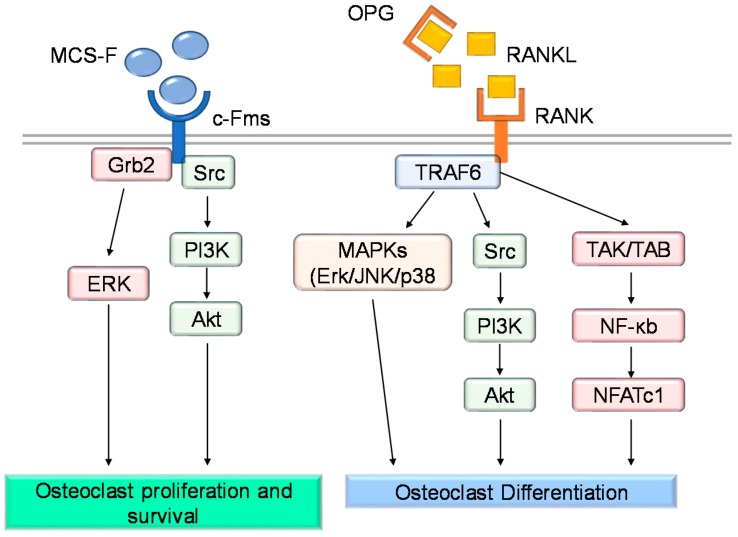
Molecular pathways involved in osteoclast proliferation, differentiation and survival. Cartoon illustrating the main intracellular pathways involved in osteoclast survival and differentiation.

Going more deeply into the molecular mechanism underlying osteoclast differentiation, the story becomes more complex (Figure 2). M-CSF enhances the proliferation and survival of preosteoclasts [17]. The understanding of its role became clear after the finding that mice lacking functional M-CSF (i.e., op/op mice), or knockout for the M-CSF receptor (csfr1^−/−^ mice), displayed an osteopetrotic phenotype due to the complete lack of osteoclasts [18]. Another important role played by M-CSF is to elicit the expression of RANK receptor by osteoclast precursors [19].

RANKL/RANK signaling is pivotal for osteoclast differentiation [20,21]. Within the bone, RANKL is mainly produced by stromal cells, osteoblasts and osteocytes [22]. Osteoblasts also produce osteoprotegerin (OPG) that acts as a decoy receptor for RANKL, inhibiting its binding to RANK expressed by the osteoclast precursors [21,23]. Thus, OPG is considered a negative regulator of osteoclast differentiation [20]. Both RANK- and RANKL-deficient mice show a severe osteopetrotic phenotype, caused by defective osteoclast formation [24,25,26]. On the other hand, a mouse model lacking OPG showed an overall reduction of the bone density due to increased osteoclast differentiation [27]. Activation of RANKL/RANK signaling induces the recruitment of TRAF6 (TNF Receptor Associated Factor 6), which in turn allows the activation of the Nuclear Factor Kappa B (NF-κB) transcription factor, which translocates into the nucleus and promotes the transcription of genes regulating osteoclast differentiation. Finally, RANKL-mediated NF-κB and c-fos activation enable the recruitment of NFATc1 (Nuclear Factor of Activated T-cells Cytoplasmic 1), which is essential for complete osteoclast differentiation [28]. NFATc1 stimulates the expression of osteoclast-specific genes, including the calcitonin receptor, TRAcP, Cathepsin K, OSCAR and β3 integrin [29,30,31,32].

Another crucial step of osteoclast differentiation is the fusion of the osteoclast precursors. This process is mediated by several molecules, including integrins, the proto-oncogene c-src, E-cadherin, DC-STAMP (dendritic cell-specific transmembrane protein), ADAM (a disintegrin and metalloproteinase) family proteins, the macrophage fusion receptor (MFR) and the V0 subunit d2 of the V-H+-ATPase [33,34,35,36,37].

#### 2.2.1. Osteoclast Function

**Figure 3:**
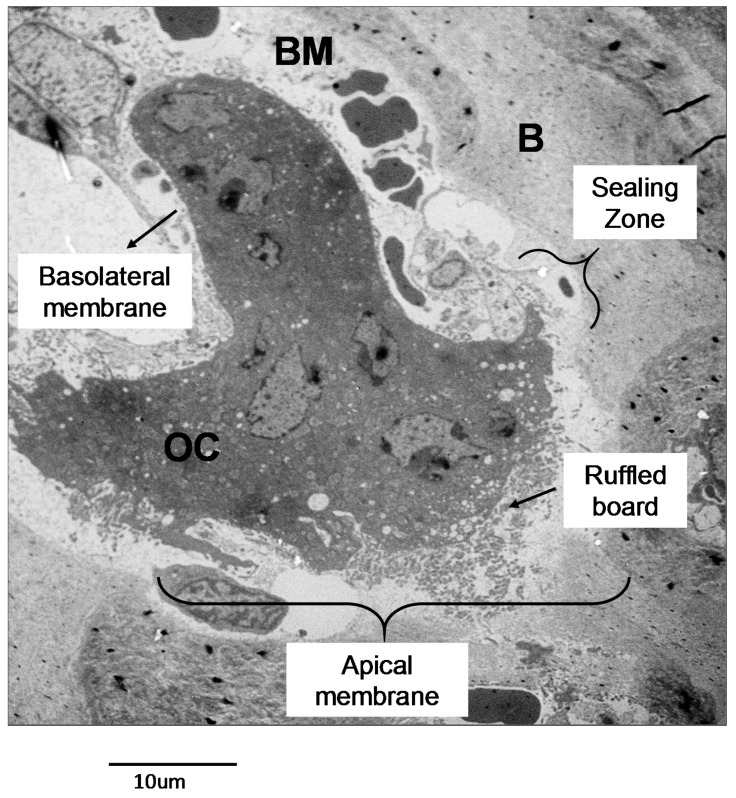
Osteoclast morphology. Transmission electron microscopy (TEM) picture of an osteoclast from an Epon-embedded tibia from a C57/B6 mouse. Scale 10 µm. B: bone. OC: osteoclast. BM: bone marrow.

A mature osteoclast is a multinucleated and polarized cell, in which it is possible to identify specific membrane domains reflecting specific functions (Figure 3). In the apical membrane there is a “sealing zone” that contains two distinct areas called the “sealing membrane” and the “clear zone” [16]. The “sealing membrane” is a bone-facing membrane portion including adhesion structures called podosomes [38]. These are made by actin microfilaments, adhesion molecules, adapter and signaling proteins, that during bone resorption are essential for the formation of peripheral hoops called actin rings [39]. Moreover, the integrin receptors α_2_β_1_, α_v_β_3_ and α_v_β_5_, anchor the osteoclast to the extracellular matrix. This tight adhesion is mandatory for osteoclast activity, thus sealing the portion of the bone matrix to be degraded. Adjacent to the “sealing zone” is the “ruffled board”, a peculiar membrane structure consisting of several membrane expansions [40]. Here, the lysosomal membranes fuse with the osteoclast membrane allowing the release of lysosomal enzymes that will digest the organic part of the matrix. Moreover, the ruffled board contains transporters essential for the release of ions, mainly chloride and protons, and for the re-uptake of the resorbed materials from the resorption lacuna. Moving further from the bone surface we encounter the basolateral membrane domain that is in contact with the vascular compartment and presents several ion transporters [41]. Recently, novel specialized subdomains have been described [42]. In particular, the ruffled board presents a “fusion zone” and an “uptake zone” that are involved in the endo/lysosome vesicular fusion, the re-uptake of digested bone matrix and recycling of the lysosomal enzymes [42,43]. Moving to the basolateral membrane, a “functional secretory domain” is present, which is essential for releasing the digested matrix components into the blood stream [43].

Once adhered to the bone surface, the osteoclast can start the process of bone resorption. This process firstly requires the acidification of the area targeted for digestion, called the resorption lacuna or Howship lacuna, since it only becomes possible to dissolve the hydroxyapatite crystals with this chemical reaction [44]. On the intracellular side, type II carbonic anhydrase (CAII) catalyzes the hydration of carbonic anhydride (CO_2_) thus forming carbonic acid (H_2_CO_3_), which in turn dissociates and releases protons (H^+^) and carbonate ions (HCO_3_^−^). The former is pumped outside the osteoclast into the Howship lacuna by the Vacuolar-ATPase (V-ATPase) proton pump inserted in the ruffled border. At the same time, the chloride ion (Cl^−^)/HCO_3_ exchanger allows the Cl^−^ to enter and HCO_3_^−^ to leave the osteoclast. Then, the proton/chloride antiporter ClC7 (chloride channel 7) also inserted in the ruffled border, together with its β-subunit OSTM1 (osteopetrosis-associated transmembrane protein 1), allows the release of Cl^−^ into the Howship lacuna [16,45]. Gene mutations in any one of the members of this molecular machinery induces the osteoclast-rich osteopetrosis [46,47], characterized by a high osteoclast number, which however are unable to resorb bone.

We now have hydrochloride acid (HCl) inside the resorption lacuna, which allows the dissolution of the inorganic matrix and the subsequent exposure of collagen fibers, which can now be digested by lysosomal enzymes, mainly acid hydrolases, released into the lacuna by the osteoclasts [16,45]. Cathepsin K (CstK) and Metalloproteinase 9 are crucial enzymes involved in this process [48,49,50].

#### 2.2.2. Regulation of Osteoclast Differentiation and Activity

Together with the M-CSF and RANKL, there is a network of paracrine and systemic factors regulating osteoclast differentiation [19,51]. Among these, the proinflammatory cytokines play a crucial role in the enhancement of osteoclast differentiation. Indeed, TNFα induces osteoclast differentiation directly by activating NF-κB and JNK (c-Jun N-terminal kinases) in a RANKL-independent manner [52] and indirectly by stimulating the osteoblasts to express RANKL [53]. In the same way, IL-1, IL-6 and IL-11 are potent osteoclastogenic factors [54,55,56,57]. Other molecules, such as EGF (epidermal growth factor), TGFα, OSF (osteoclast-stimulating factor), ECF-L (eosinophil chemiotactic factor-L) and Activin A increase both osteoclast differentiation and activity [54,58,59,60].

**Figure 4:**
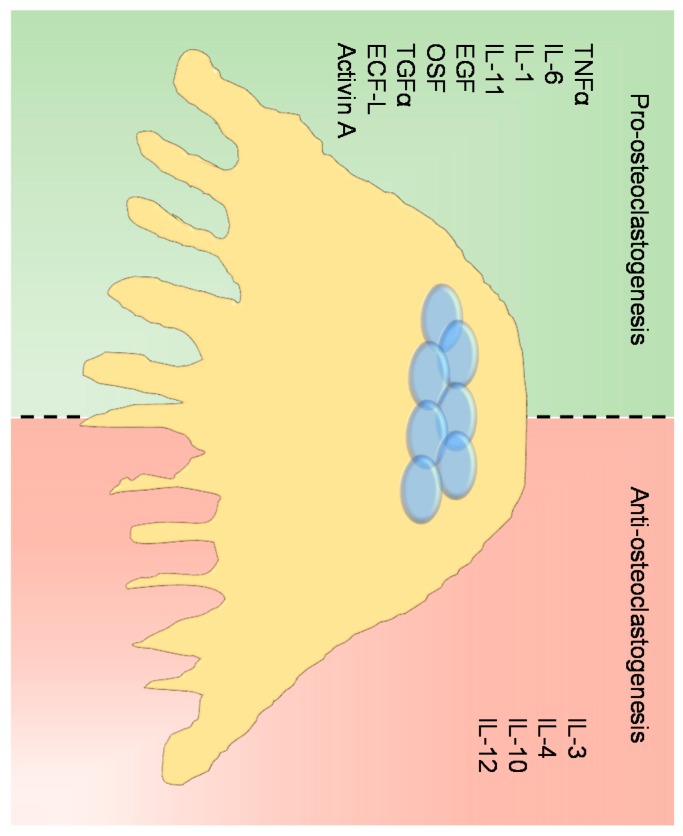
Osteoclast regulation. Cartoon illustrating the main molecules involved in the regulation of osteoclast differentiation with a pro- (green side) and anti- (red side) osteoclastogenic effect.

In contrast, in addition to OPG, cytokines such us IL-3, IL-4, IL-10 and IL-12 inhibit osteoclast differentiation and bone resorption [61,62,63,64] (Figure 4).

Several hormones regulate osteoclast fate, including estrogen and testosterone, PTH, calcitonin, glucocorticoids, thyroid hormone and serotonin [51,65]. According to the topic of this review article we will focus our attention on the ability of sex hormones to regulate osteoclast activity.

Both estrogens and androgens impair osteoclast formation and survival [51,66]. Sex hormones can act through a genotropic signal, by binding their intracellular receptor and, after translocation into the nucleus, inducing the transcription of target genes. They can also act through a non-genotropic pathway mediated by plasma membrane receptors eventually leading to the stimulation of calcium flux and the activation of cytoplasmic kinases [51,66].

It has been demonstrated that estrogens induce the expression of the death receptor First Apoptosis Signal (FAS)-ligand in osteoclasts, thus acting as a pro-apoptotic stimulus [67]. Indeed, the lack of ERα does not affect osteoclast differentiation, however it increases their lifespan [67].

## 3. Alterations of Bone Remodelling: The Vicious Cycle

**Figure 5:**
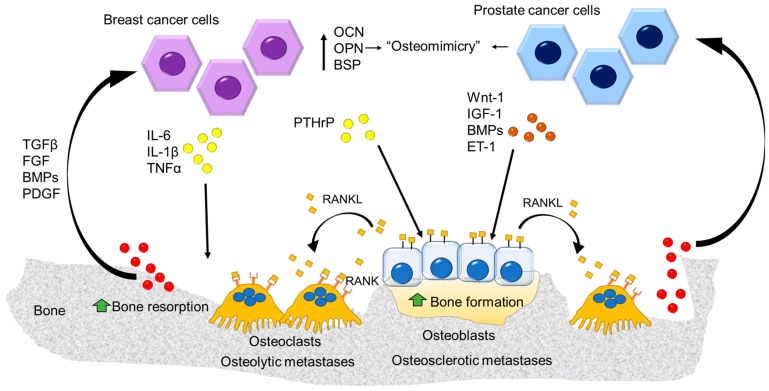
The “vicious” cycle. Breast cancer cells release several factors stimulating osteoclast formation and enhancing bone resorption (osteolytic metastasis). On the other side, the prostate cancer cells act on the osteoblast stimulating bone formation (osteosclerotic metastasis) along with the stimulation of the osteoblast-mediated osteoclastogenesis via RANKL expression inducing osteolytic lesions. At the same time the factors released from the bone matrix enhance the tumor growth fueling the “vicious” cycle.

As already told, any unbalance between the resorption and formation phases is responsible for an altered bone mass, eventually leading to a pathological condition. Regardless of the type of bone metastases (i.e., osteolytic vs. osteosclerotic), they are the result of a severe deregulation of the bone remodeling process, thus transforming a virtuous cycle into a vicious one (Figure 5).

**Figure 6:**
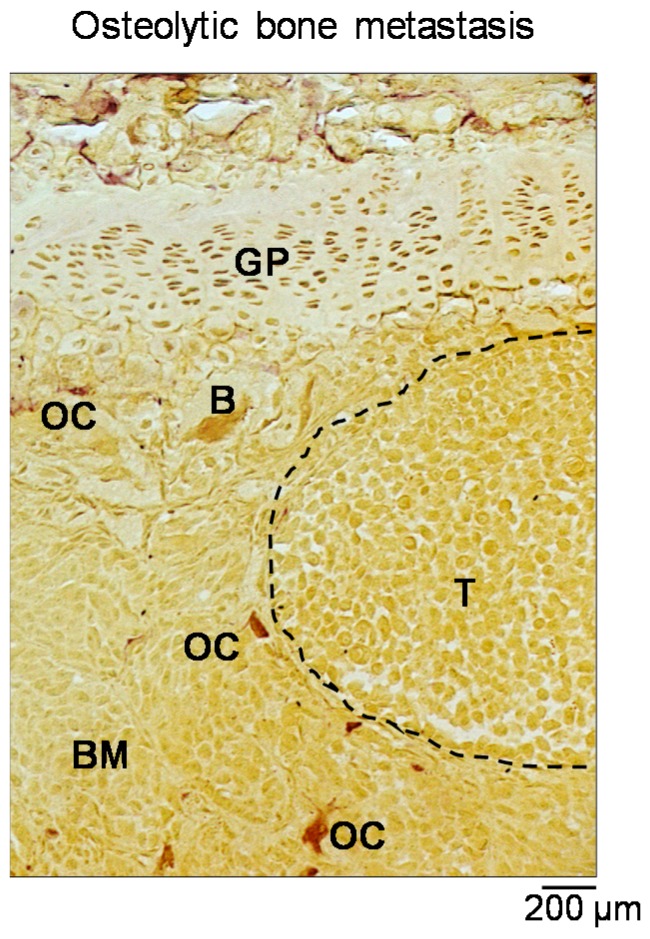
Osteolytic bone metastasis. Staining for the tartrate-resistant acid phosphatase (TRAcP) activity perfomed on a histological section of a tibia collected from a BALB-c nu/nu mouse intratibially injected with the human breast cancer cells MDA-MB-231. Purple cells represent the osteoclasts. Dashed line: tumor area. Scale: 200 µm. B: bone. OC: osteoclast. BM: bone marrow. GP: growth plate. T: tumor.

The vicious cycle is the core of bone metastasis development and strictly relies on the ability of tumor cells to “subjugate” bone resident cells (i.e., osteoblasts and osteoclasts) for their own benefit. Tumor cells able to reach the bone present with specific features, enabling their survival in a hostile tissue. One of these is summarized by the phenomenon of “osteomimicry”, that is the ability of tumor cells that preferentially metastasize the bone to express a genetic profile resembling that of the resident cells, thus acquiring a more favorable phenotype for survival in the bone [68]. More specifically, we refer to an osteoblast mimicry, by which both prostate and breast cancer cells produce bone matrix proteins such as osteocalcin (OCN), bone sialoprotein (BSP) and osteopontin (OPN) which are also involved in tumor invasion [69,70,71]. Since osteoblasts are the main paracrine regulators of osteoclasts in the bone, this function is also included in the osteomimetic properties of tumor cells [9]. In the case of osteolytic bone metastases (Figure 6), tumor cells produce several factors that directly or indirectly account for an exacerbated formation and activity of osteoclasts. The former include several cytokines, such as IL-6, IL-1β and TNFα, all promoting the formation of an abnormal number of osteoclasts, eventually leading to an exaggerated bone erosion which would never have been balanced by a proper bone formation. Of note, these factors also act on osteoblasts by promoting their apoptosis [72]. This scenario, compatible with a condition of chronic inflammation, further highlights the role of this condition in tumor progression and metastasis.

Another crucial molecule in this vicious cycle is the parathyroid hormone-related protein (PTHrP), a paracrine regulator of bone remodeling produced by osteoblasts. Tumor cells indirectly stimulate osteoclastogenesis through this molecule as it increases RANKL expression by osteoblasts [73].

The exaggerated osteoclast formation and bone destruction create the physical space in which tumor cells can grow and reach a critical mass. However, this huge destruction of bone matrix also leads to another nasty consequence which is responsible for the perpetuation of the vicious cycle: the release of previously mentioned growth factors, such as BMPs, TGFβ, FGF, and platelet–derived growth factor (PDGF). In physiological condition, the factors help osteoclast–osteoblast factor coupling, whereas in this circumstance, they exert a prosurvival effect on the tumor cells.

Another detrimental effect of the vicious cycle is the release of an abnormal amount of calcium, responsible for hypercalcemia, which dramatically worsens the condition of bone metastatic patients. Likewise, a direct effect of calcium on tumor cells has also been demonstrated, as the cells can express calcium-sensing receptors, eventually leading to an increased proliferation and survival of tumor cells [74].

A similar vicious cycle is also established in osteosclerotic lesions, although in this case the prominent players are tumor-derived molecules affecting osteoblast differentiation and function [75]. Among them, wingless-related integration site (Wnt)-1, IGF-1, BMPs and endothelin (ET)-1 are the main molecules secreted by prostate cancer cells that stimulate the osteoblasts [76,77]. These factors induce the deposition of new unmineralized bone matrix (osteoid) enriched in growth factors and non-collagenous proteins. This fertile ‘soil’ attracts the prostate cancer cells, allowing them on one side to growth and survive in the bone environment, and on the other to stimulate the osteoblasts, thus enhancing the vicious cycle [78,79]. Paradoxically, the increased bone formation could reduce the space for the tumor cells in the bone environment, slowing down the progression of the osteosclerotic lesion. Nevertheless, simultaneously factors produced by the prostate cancer cells and the osteoblast-derived RANKL stimulate bone resorption creating the space for the tumor growth [75,78,79]. Once the disease enters the ‘osteolytic phase’ the osteoclast activity becomes prominent, thus reducing patient survival [75,78,79].

Recently, new findings demonstrated that the vicious cycle in prostate cancer-induced bone metastases is a complex system. Indeed, several players including exosomes, mesenchymal stem cells, macrophages, T cells and nerves have been indicated as integrated parts of the vicious cycle in osteoblastic skeletal lesions [80].

Once again, this evidence highlights the complexity of the bone metastases and explains why their treatment represents a real challenge for the researchers in the field.

## 4. The Osteoclast as Therapeutic Target

Since the exacerbated osteoclast activity is a key point common to all bone metastases, no matter if they are osteolytic or osteosclerotic, it is conceivable to identify the osteoclast as a very useful target. Over the years, the physiology of the osteoclast has been deeply investigated; now we have quite a complete picture of the most important mechanisms underlying its formation and activity. This leads us to the identification of useful targets aimed at restraining the exacerbated bone destruction occurring in bone metastases. In line with this, several antiresorptive molecules have been identified so far, which are frequently successfully used for the treatment of post-menopausal osteoporosis.

Below we will describe the principal therapeutic arms employed so far, as well as some new promising therapeutic strategies under preclinical investigation.

### 4.1. Current Antiresorptive Therapies

#### 4.1.1. Bisphosphonates

It seems unbelievable, but these compounds were synthesized for the first time at the end of the XIX century for purposes not related at all to bone health. They were used in the detergent industry (i.e., acid etidronic, or etidronate) and for the depuration of aquifer, thanks to their phosphate-carbon-phosphate (P-C-P) backbone structure, by which they efficaciously chelate calcium and some ion metals. Due to the high affinity for hydroxyapatite crystals, the use of bisphosphonate therapeutics in osteoporosis started 40 years ago, giving rise to different generations of bisphosphonates, all targeting the osteoclast, until the third-generation compound zoledronate. Once delivered to the bone, they are taken up by the osteoclasts where they induce apoptosis by different mechanisms [81]. Clodronate and etidronate belong to the non-nitrogen containing bisphosphonates, which induce osteoclast apoptosis by inhibiting the mitochondrial adenine nucleotide translocase [82]. Nitrogen containing bisphosphonates (i.e., ibandronate, pamidronate, risedronate, alendronate and zoledronate) induce osteoclast apoptosis by interfering with the mevalonate pathway, leading to the accumulation of triphosphoric acid 1-adenosin-5′-y1 ester 3-(3-methylbut-3-enyl) ester (ApppI) and of isopentenyl pyrophosphate (IPP), which in turn induce osteoclast apoptosis [83]. Moreover, the nitrogen-containing bisphosphonates inactivate osteoclasts by inhibiting the enzyme farnesyl diphosphate synthase in the cholesterol pathway, leading to a reduction of GTPase prenylation, an essential process for the cytoskeletal organization and vesicular trafficking [84].

Currently, the most effective bisphosphonate seems to be zoledronate, which significantly prevents skeletal related events and improves the quality of life [85]. Interestingly, in clinical trials in which zoledronate has been used in adjuvant and neoadjuvant therapy of early breast cancer patients, it has also been shown to have anticancer activity, specifically in those patients with low levels of estrogens, eventually leading to improved survival [86].

Unfortunately, bisphosphonates can have some side effects such as osteonecrosis of the jaw, bone/joint pain and atypical fractures [87]. Moreover, long term treatments can cause hypocalcemia [88]. Another pitfall of this treatments is caused by the high affinity to the bone: bisphosphates build up in this tissue and remain for a long time, causing the bone to become adynamic.

#### 4.1.2. Denosumab

This is a humanized anti-RANKL antibody, which blocks osteoclast formation by inhibiting RANKL-RANK interaction. The drug reflects the physiological role of OPG; the recombinant OPG-Fc protein was first synthesized to block the RANKL/RANK pathway in preclinical studies. This compound was then discontinued for its adverse effects linked to an immune response to OPG-Fc [86].

Phase-III clinical trials demonstrated Denosumab to be more effective in delaying the onset of SREs and time to first bone metastases in prostate and breast cancer patients compared to Zoledronate, eventually leading to an increase of bone metastasis-free survival [85].

The incidence of hypocalcemia in Denosumab-treated patients is higher than with zoledronate treatment (12.4% vs. 5.3%, respectively) [88]. By contrast, zoledronate is excreted by the kidney and therefore should not be recommended to prevent SREs in patients suffering from renal failure. These patients could instead benefit from treatment with denosumab, which is not cleared by the kidney [89]. Other side effects reported are: osteonecrosis of the jaw, nausea, and fatigue [85].

### 4.2. Anti Resorptive Treatment as an Adjuvant Therapy in Breast Cancer Patients

Neoadjuvant treatment of breast cancer patients with anti-estrogen therapies, despite reducing tumor cell proliferation, is instead detrimental for bone, giving rise to a condition of osteoporosis and increased incidence of fracture. Most importantly, this condition likely creates favorable conditions for the development of osteolytic bone metastases. Therefore, it is now clear that there is an urgent need to preserve bone health in breast cancer patients. According to the recent guidelines of the European Society for Medical Oncology, breast cancer patients should be monitored for their bone mineral density. Moreover, some reports suggest that for those breast cancer patients who are at risk of cancer treatment-induced bone loss, which includes not only the aromatase inhibitors but also some chemotherapies (i.e., Doxorubicin, 5-fluorouracil), a preventive treatment with zoledronate or denosumab could be beneficial to improve survival. Indeed, there is evidence that early intervention may delay the development of bone metastases. Analysis of patients with breast cancer and bone metastases using data from two phase II clinical trials revealed that the benefit of high-dose zoledronic acid was greater in patients whose therapy was initiated before the onset of bone pain than in those who received treatment after the onset of bone pain [6]. Moreover, bone metastases were more common in women who received delayed treatment than in those who started zoledronic acid immediately [5].

### 4.3. Antitumoral Effects of Antiresorptive Therapies

Some in vitro and preclinical studies highlighted a role for zoledronate and denosumab as inhibitors of tumor growth. This is obvious for denosumab, since RANKL plays a key role in mammary gland development as well as in its tumorigenesis [90,91]. In addition, the inhibition of the effect of RANKL could enhance the antitumor immune response, thus reducing the regulatory T cells [89]. To support the role of RANKL in tumor progression, patients with low or no expression of RANK in primary lesions showed a better survival outcome [92].

With regard to bisphosphonates, recent evidence shows that zoledronate induces caspase-dependent apoptosis in renal cancer, impairs angiogenesis and reduces the migration and invasion of tumor cells [93,94,95]. Moreover, zoledronic acid induces modulations of the immune system increasing the sensitivity of tumor cells to the γδ T cell-mediated cell lysis [95].

Finally, clinical data revealed that the use of bisphosphonates was correlated with a reduction in risk of developing postmenopausal breast cancer [96,97] along with a reduction of breast cancer-associated mortality [98].

The inhibitory effect of bisphosphonates, particularly zoledronate, on tumor progression and dissemination may be explained at least in part by the fact that they are able to change the bone microenvironment making it less prone ‘to host’ and support the tumor cells [99].

### 4.4. New Antiresorptive Therapeutic Strategies

Recent studies have been focused on identifying new alternative therapies fighting the vicious cycle by targeting the osteoclasts [86], as described below.

#### 4.4.1. Cathepsin K Inhibitors

Cathepsin K is a lysosomal cysteine protease highly expressed by osteoclasts. Following mineral matrix dissolution, cathepsin K degrades the collagen fibers, leading to the release of N-telopeptides fragments [16]. In the context of bone metastasis, cathepsin K is expressed by osteoclasts and some metastatic breast cancer cells [100]. However, the inhibition of cathepsin k does not affect the tumor cell proliferation [101].

Although results of in vitro and preclinical studies aimed at inhibiting cathepsin K activity were promising, all clinical trials for cathepsin K inhibitors have been discontinued. In particular, the basic lysosomotropic cathepsin K inhibitor Balicatib (clinical phase II; NCT00371670) was able to reduce bone resorption increasing the BMD in patients with osteoarthritis. Nevertheless, its clinical development has been discontinued due to its side effects on skin, caused by a prolonged lysosomal trapping of the inhibitor [102].

Another promising cathepsin K inhibitor was Odanacatib. Indeed, pre-clinical and clinical studies showed that it was effective in treating both osteoporosis and cancer-associated bone metastases with the same efficacy as zoledronate [103]. Unfortunately, Odanacatib was also discontinued in phase III clinical trials due to an increased risk of atrial fibrillation and stroke.

#### 4.4.2. c-src Inhibitors

As already mentioned, the proto-oncogene c-src plays a relevant role in bone metabolism. In fact, c-src knock out mice present with an osteopetrotic phenotype due to failure of src-mutant osteoclasts to form the ruffled border [33], while decreased c-src expression enhances osteoblast differentiation and bone formation [104]. Due to its oncogenic nature, c-src also stimulates cancer cell proliferation and tumorigenesis [105,106,107]. All these data make c-src the perfect target to block the vicious cycle.

There are several c-src inhibitors that have been developed so far, such as Dasatinib, Bosutinib, Ponatinib and Vandetanib, which are FDA-approved for different diseases. Preclinical studies carried out on metastatic prostate cancer cells showed that the c-src inhibitors Bosutinib and Saracatinib affect tumor cell proliferation and reduce cancer-induced osteolysis by inhibiting the phosphorylation of AKT, MAPK and FAK [108,109]. Moreover, Maroni et al. [110] showed that treatment with the c-src inhibitor Dasatinib increased the survival of mice xenotransplanted with the human bone metastatic 1833 cells, by the inhibition of cell autophagy.

To date there are ongoing phase I/II clinical trials for the use of Dasatinib and Saracatinib to treat bone metastasis that are promising in terms of reduction of disease progression [86].

#### 4.4.3. Inhibitors of Integrins

As already discussed in this review, integrins, and in particular αvβ3 integrin, play a central role in osteoclast biology and bone resorption. Overexpression of the αvβ3 integrin in tumor cells is also essential for their bone homing [111]. 

In vitro studies demonstrated that the anti-αvβ3 integrin antibody was able to reduce bone resorption, impairing osteoclast adhesion to the bone surface [112]. In line with this, the use of PSK1404, an antagonist of the αvβ3 integrin, in an in vivo model of breast cancer, reduced bone resorption by decreasing the secretion of pro-osteoclast activity tumor factors [113].

So far, there is an ongoing phase II clinical trial for the treatment of metastatic prostate cancer patients using the anti-αvβ3 integrin human monoclonal antibody, Etaracizumab, in combination with docetaxel, zoledronate and prednisone (NCT00072930). Unfortunately, the results of the trial are not yet public.

## 5. Anabolic Treatments

### Antisclerostin Antibodies

Sclerostin (SOST) is a protein, mainly produced by the osteocytes in bone, that binds LRP5/6 (low-density lipoprotein receptor-related protein 5 and 6) inhibiting the Wnt signaling pathways, which in turn plays a crucial role in osteoblast differentiation [114]. The inhibition of SOST has an osteoanabolic effect, inducing an increase in bone formation [115]. Interestingly, SOST is also produced by some tumor cells, including breast cancer cells, and its serum level is increased in some breast cancer patients [116,117]. Romosozumab, Blosozumab and BPS804 are human monoclonal antibodies against SOST in clinical development for the treatment of bone diseases such as osteoporosis and osteogenesis imperfecta [118,119]. Moreover, in vivo experiments performed with animal models of multiple myeloma treated with an anti-SOST antibody revealed the efficacy of this treatment in increasing bone mass and preventing tumor-associated bone loss [120]. Based on this available information, the use of anti-SOST antibodies for the treatment of bone metastasis could be taken into account.

## 6. Conclusions

For breast and prostate cancer patients, bone is the preferential site of metastases, which offers a better chance of survival compared to metastases in soft tissues. This encourages researchers in the field to identify new effective therapies to prolong life expectancy and quality of life for patients. Other strategical therapeutic options to be considered are those able to preserve bone health, a noteworthy aspect in breast and prostate cancer patients if we consider that they are treated with therapies known to inhibit sexual hormones. This is the right method to kill breast and prostate cancer cells however also having a detrimental effect on bone, thus inducing a waste in bone mass. The latter condition could create a good environment for the onset of bone metastases, therefore preserving the correct bone mass in breast and prostate cancer patients could pose an advantage, eventually preventing or delaying the occurrence of the first bone metastasis as well as the skeletal related events. A promising option could be to employ the antiresorptive treatments as early as possible in order to prevent bone loss caused by the neoadjuvant therapy.
